# Pediatric Invasive Pneumococcal Disease Caused by Vaccine Serotypes following the Introduction of Conjugate Vaccination in Denmark

**DOI:** 10.1371/journal.pone.0051460

**Published:** 2013-01-24

**Authors:** Zitta B. Harboe, Palle Valentiner-Branth, Helene Ingels, Jeppe N. Rasmussen, Peter H. S. Andersen, Catherine C. Bjerre, David Goldblatt, Lindsey Ashton, Mitch Haston, Helle B. Konradsen, Lotte Lambertsen, on behalf of the Danish Pneumococcal Surveillance Collaborating Group

**Affiliations:** 1 Neisseria and Streptococcus Reference Center, Department of Microbiology and Infection Control, Statens Serum Institut (SSI), Copenhagen, Denmark; 2 Department of Infectious Disease Epidemiology, Statens Serum Institut (SSI), Copenhagen, Denmark; 3 Immunobiology Unit, University College London Institute of Child Health, London, United Kingdom; Health Protection Agency, United Kingdom of America

## Abstract

A seven-valent pneumococcal conjugate vaccine (PCV7) was introduced in the Danish childhood immunization program (2+1 schedule) in October 2007, followed by PCV13 starting from April 2010. The nationwide incidence of IPD among children younger than 5 years nearly halved after the introduction of PCV7 in the program, mainly due to a decline in IPD caused by PCV7-serotypes. We report the results from a nationwide population-based cohort study of laboratory confirmed IPD cases in children younger than 5 years during October 1, 2007 to December 31, 2010 and describe the characteristics of children suspected to present with a vaccine failure. The period between April 19 and December 31, 2010 was considered a PCV7/PCV13 transitional period, where both vaccines were offered. We identified 45 episodes of IPD caused by a PCV7 serotype (23% of the total number) and 105 (55%) caused by one of the 6 additional serotypes in PCV13. Ten children had received at least one PCV7 dose before the onset of IPD caused by a PCV7 serotype. Seven children were considered to be incompletely vaccinated before IPD, but only three cases fulfilled the criteria of vaccine failure (caused by serotypes 14, 19F and 23F). One case of vaccine failure was observed in a severely immunosuppressed child following three PCV7 doses, and two cases were observed in immunocompetent children following two infant doses before they were eligible for their booster. None of the IPD cases caused by the additional PCV13 serotypes had been vaccinated by PCV13 and there were therefore no PCV13-vaccine failures in the first 8-months after PCV13 introduction in Denmark.

## Introduction

A seven-valent pneumococcal conjugate vaccine (PCV7) was introduced in the Danish childhood immunization program in October 2007. Shortly after its introduction, the incidence of invasive pneumococcal disease (IPD) declined markedly, in particular among children younger than 2 years, where the incidence halved compared to the pre-vaccination period [1], [2]. The decline was mainly related to a decrease in the incidence of IPD caused by vaccine serotypes (VT-IPD) [2]. These results are consistent with those observed in other industrialized countries and support the high effectiveness of PCV7 against VT-IPD [3]–[6].

Changes in the serotype distribution of invasive pneumococci were described to occur at an early stage in the post-vaccination period in Denmark and non-vaccine serotypes 7F and 1 were found predominantly as a cause of non-VT-IPD in children [7]. However, and similarly to what has been observed in other countries, cases of VT-IPD are still observed in the population during the post-PCV7 period among children for whom vaccination was offered [8], [9].

Cases of VT-IPD raise concerns related to the vaccine's effectiveness and to the possibility of immunological disorders in the affected child. It is well recognized that not all serotypes in the pneumococcal conjugate vaccines (PCV) are equally immunogenic and that the immune response also varies according to the number of doses administered [10]–[12]. Furthermore, incomplete vaccination and co-morbid conditions (such as immunodeficiencies, malignancies, prematurity, and several chronic diseases, among others) have been identified as factors contributing to VT-IPD in vaccinated children [8], [9]. Also, cases of vaccine failure in apparently immunocompetent children have been described [13]–[15].

In October 2007, an enhanced nationwide, population-based surveillance of laboratory confirmed IPD cases in children younger than 5 years was implemented in Denmark in order to identify patients where a vaccine failure could be suspected. This information is considered valuable in order to understand the underlying mechanisms behind a case of vaccine failure and may contribute to identify a subpopulation of children that might need additional immunological investigation.

We hereby report the results from a nationwide population-based cohort study of laboratory confirmed IPD cases in children younger than 5 years of age and describe the characteristics of children with vaccine-type IPD during a 31-month surveillance period.

## Materials and Methods

### Study Setting and Design

We conducted a nationwide cohort study based on population-based surveillance data on laboratory confirmed IPD in children younger than 5 years of age after the introduction of PCV in the Danish childhood immunization program. Laboratory surveillance data on IPD from October 1, 2007 to December 31, 2010 were linked to the Danish Childhood Vaccination Registry as previously described [2]. Information regarding the patients' clinical course and blood samples for immunological testing were routinely collected by a physician in cases where a vaccine failure was suspected. Since data and samples from patients were collected routinely for national surveillance purposes, no ethical approval or informed consent from patients were required. The study was approved by the Danish Data Protection Agency (no. 2007-41-0229).

### Pneumococcal Conjugate Vaccination in Denmark

A PCV has been offered free of charge as a part of the routine childhood immunization program since October 1, 2007 in a reduced 2+1 schedule at the ages of 3, 5, and 12 months. PCV7 started to be offered from October 1, 2007. For an introduction period, children aged 12–17 months at the first vaccination (born after April 30, 2006) were offered a catch-up program of two doses of PCV7 with a minimum interval of two months [16]. The thirteen-valent pneumococcal conjugate vaccine (PCV13) started to be offered on April 19, 2010 [17]. General practitioners were recommended to use their stocks of PCV7 before start using PCV13, and therefore the period following the introduction of PCV13 can only be considered as a transition period, where both PCV7 and PCV13 were administered.

During 2007–2010, approximately 64.000 children were born annually in Denmark (http://www.statistikbanken.dk). The vaccination uptake of PCV7 and the concomitantly administered single shot vaccine against diphtheria, tetanus, acellular pertussis, poliomyelitis and *Haemophilus influenzae* type b (DTaP-IPV/Hib Statens Serum Institut, Copenhagen, Denmark) has been reported elsewhere [1], [18]. In brief, PCV7's uptake was reported to be approximately two percentage points lower than the uptake of DTaP-IPV/Hib vaccine. The uptake of the primary vaccination program with PCV7 for the birth cohorts 2007–2009 was 81–91% for the first, second and booster doses. The uptake of the catch-up program was lower, 51–76% depending on the age of the child and the number of doses given [18]. No shortages in stockpiles or interruption of the distribution of the vaccines have been reported.

### National Surveillance of Pediatric IPD and PCV-Failures

**Figure 1:**
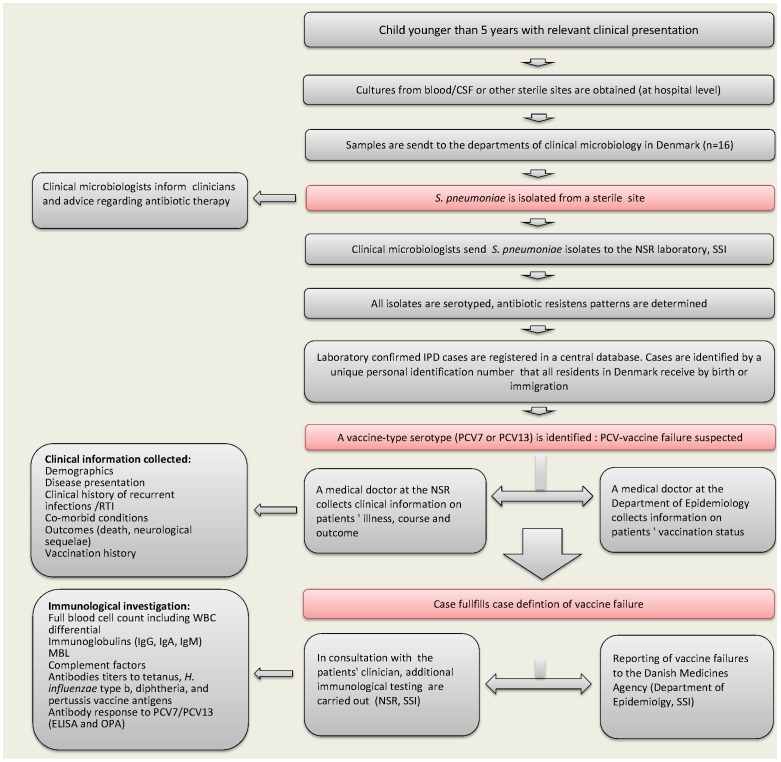
Danish surveillance system of pediatric invasive pneumococcal disease (IPD) and pneumococcal conjugate vaccine (PCV)-failures. Cases of IPD caused by a serotype included in PCV7 or one of the additional serotypes included in PCV13 are identified among all IPD cases in children <5 years in order to explore possible cases of vaccine failure. PCV7 was administered in the national childhood immunization program free of charge from October 1, 2007 to April 19, 2010 and then PCV13 was offered. *CSF*: Cerebrospinal fluid; *NSR:* National Neisseria and Streptococcus Reference Center, Statens Serum Institut: SSI, *MBL:* Mannose-Binding Lectin, *RTI:* Respiratory Tract Infections, *WBC:* White Blood Cells.

Figure 1 shows the components and setup of the national surveillance system of pediatric IPD and PCV-failures. In brief, all departments of clinical microbiology in Denmark send invasive *Streptococcus pneumoniae* isolates for determination of serotype to the National Neisseria and Streptococcus Reference Center (NSR), Statens Serum Institut (SSI). The characteristics and completeness of the national surveillance system for IPD have previously been described [19]. Approximately 90% of the isolates are obtained from hospitalized patients [20]. Notification of IPD in children younger than 5 years of age and submission of pneumococcal isolates causing IPD in all age groups became mandatory by law after October 1, 2007 in order to monitor the effectiveness of PCV [21].

Children who present with IPD due to one of PCV7-serotypes or one of the six additional serotypes included in PCV13 (after PCV13 became available in Denmark) are considered suspects of having a vaccine failure. PCV7 contains the capsular polysaccharide antigens of 7 serotypes (4, 6B, 9V, 14, 18C, 19F, and 23F). Serotypes 1, 3, 5, 6A, 7F, 19A are the six additional serotypes included in PCV13. Clinical information and vaccination status of patients are collected by telephone interview with clinicians in charge of the patient and by verification of discharge diagnoses and medical files (Figure 1). During the transition period when both PCV7 and PCV13 were administered, the vaccine's batch number was used to verify the valence of the vaccine administered to the child, if relevant. If a case fulfills the case definition of vaccine failure, supplementary blood samples from patients are requested for additional immunological investigation, in consultation with the patient's physician (Figure 1). Cases of VT-IPD in vaccinated children, regardless of the number of vaccines they had received, are reported to the Danish Medicines Agency (Lægemiddelstyrelsen) by the Department of Infectious Disease Epidemiology, SSI.

### Case Definitions

#### Invasive Pneumococcal Disease (IPD)

A case of IPD is defined as the occurrence of IPD confirmed by a positive culture for *S. pneumoniae* from a patient's cerebrospinal fluid (CSF), blood or other sterile site. When both CSF and blood isolates are received from a patient, the case is categorized as meningitis. Patients are considered having a recurrence if a new IPD isolate is obtained within 30 days after the initial incidence, or if a new pneumococcal serotype is isolated within 30 days.

#### Non-vaccinated, Non-eligible

Children outside the age group targeted for primary PCV7/PCV13 vaccination or catch-up program with PCV7.

#### Non-vaccinated, Eligible

Children within the age group targeted for primary PCV7/PCV13 vaccination or catch-up program who presented an IPD episode before vaccination.

#### PCV7-Vaccinated

Children who presented an IPD episode after PCV7 immunization, regardless of the number of doses received, the time of onset of illness after vaccination and the age of immunization.

#### PCV-Failure

Defined as IPD caused by any of the serotypes included in all the vaccine- doses the child has received, and where one of the following criteria are met: 1) the child is under 13 months of age at IPD onset and has received two doses of PCV7 or PCV13 but not yet the booster dose, and becomes ill more than two weeks after the last dose was given; 2) the child is at least 12 months old at IPD onset, completed the primary vaccination before 12 months of age, and becomes ill more than one week after administration of the booster dose 3) the child is at least 6 months old at IPD onset, received two doses of PCV7 as a part of the catch-up program and becomes ill more than two weeks after the administration of the last dose. Children included in the first two categories were targeted during their primary vaccination and children in the third category were targeted by the catch-up program.

#### IPD-related Mortality

Vital status of children where PCV-failure was confirmed is retrieved through the surveillance system. Death is considered to be related with IPD when the date of death is registered within 30 days after the clinical sample was obtained from the patient.

### Laboratory Investigations

#### Pneumococcal Serotyping

All IPD isolates received are routinely serotyped by using pneumotest latex and/or Quellung reaction using type-specific pneumococcal rabbit-antisera from SSI (SSI-Diagnostica, Copenhagen, Denmark) [2], [22], [23].

#### Serological Testing

In cases that fulfilled criteria for PCV-failure, blood samples are requested in order to characterize the patient's antibody response after vaccination and infection. Serum samples are assayed for individual antibodies included in PCV7 or PCV13 according to the immunization schedule. Samples are analyzed by ELISA after absorption with polysaccharide 22F and cell wall polysaccharide and results expressed as concentrations of antibody against each PCV serotype. A serotype specific antibody value of 0.35 µg/mL is considered protective. ELISA assays are carried out at the Pneumococcal Serology Laboratory (SSI) and in this study, at the WHO reference laboratory for Pneumococcal Serology at the UCL Institute of Child Health, London, as previously described [24]–[26]. Functional activity of specific pneumococcal antibodies (PCV7 antibodies+6A) was measured by a opsonophagocytic assay (OPA) at the laboratories in London [27]. Additional immunological testing is carried out according to the child's clinical presentation (Figure 1).

## Results

**Figure 2:**
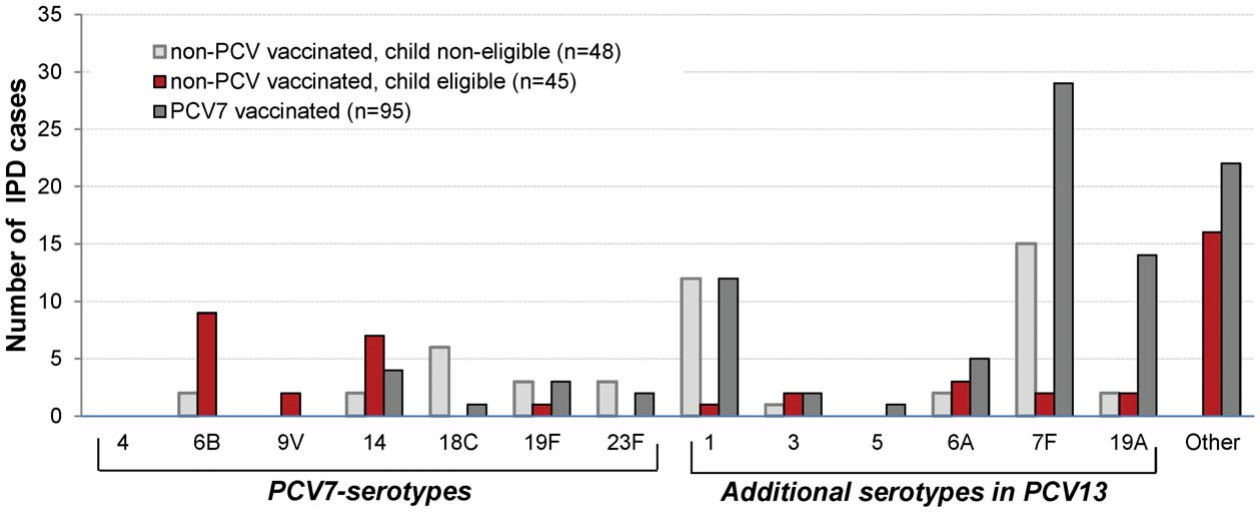
Serotype distribution of cases of Invasive pneumococcal disease in children <5 years, Denmark 2007–2010 (n = 191). Information on pneumococcal serotypes was available for all cases, but not for three cases diagnosed by PCR (not shown in the figure). Vaccination status was available for all patients but one (IPD due to serotype 35F).

From October 1, 2007 to December 31, 2010, 191 laboratory-confirmed IPD cases in 188 children younger than 5 years were reported, corresponding to an approximate estimated incidence of 14.6 (95% confidence interval (CI) 12.6–16.7) per 100,000 population (Table 1, Figure 2). The median age of children was 20 months (age range 0–59 months), 80% (n = 154) of isolates were obtained from blood, 20% (n = 36) CSF and only one isolate from other sterile sites (<1%). Pneumococcal serotype and vaccination status was available for all patients except in one, presenting with an IPD due to serotype 35F.

**Table 1:** Invasive Pneumococcal Disease in Children Younger than 5 years after the introduction of the Heptavalent Pneumococcal Conjugate Vaccine in Denmark.

PCV7 serotypes	Non-Vaccinated		PCV7-vaccinated^3^	Total
	Eligible^1^	Non-eligible^2^		
***4***	0	0	0	**0**
***6B***	9	2	0	**11**
***9V***	2	0	0	**2**
***14***	7	2	4	**13**
***18C***	0	6	1	**7**
***19F***	1	3	3	**7**
***23F***	0	3	2	**5**
***all***	*19 (42%)*	*16 (36%)*	*10 (22%)*	***45 (100%)***
**Additional serotypes in PCV13**				
***1***	1	12	12	**25**
***3***	2	1	2	**5**
***5***	0	0	1	**1**
***6A***	3	2	5	**10**
***7F***	2	15	29	**46**
***19A***	2	2	14	**18**
***all***	*10 (10%)*	*32 (30%)*	*63 (60%)*	***105 (100%)***
**Other serotypes** 4	*16 (42%)*	*0*	*22 (58%)*	***38 (100%)***
**PCR**	2 (67%)	1 (33%)	0	**3** ***(100%)***
**Total**	**47**	**49**	**95**	**191**

1 ***Eligible:*** child within the age group targeted for primary PCV7/PCV13 vaccination or catch-up program who presented an IPD episode before vaccination.

2 ***Not-eligible:*** child outside the age group targeted for primary PCV7/PCV13 vaccination or catch-up program with PCV7.

3 ***PCV7-vaccinated:*** children who presented an IPD episode after PCV7 immunization, regardless of the number of doses received, the time of onset of illness after vaccination and the age of immunization.

4 ***Other serotypes:*** 6C, 8, 10B, 12F, 15B/C, 16F, 18F, 22F, 23F, 24F, 33F, 35B, 35F. No clear predominance of any serotype was observed. None of the patients presenting IPD due to PCV7 or PCV13 serotypes were vaccinated with PCV13.

We identified 45 IPD episodes (23% of the total number) caused by a PCV7 serotype and 105 (55%) caused by one of the 6 additional serotypes in PCV13 (Table 1). None of the patients presenting IPD due to PCV7 or one of the 6 additional serotypes in PCV13 were vaccinated with PCV13. The most frequent situation was a case of IPD caused by one of the additional serotypes included in PCV13 in a child who had received at least one dose of PCV7 since the beginning of the immunization program. Among these patients, the most frequently isolated serotype was serotype 7F, 19A and 1. Among cases of non-PCV7/PCV13 serotypes after PCV13 started to be used, we identified two cases of 12F-IPD, two cases of 24F-IPD and one case caused by 33F serotype, All these patients received at least 2 doses of PCV7 before IPD.

Three patients (1.6%) presented a recurrent IPD episode: one child presented a 16F and a serotype 14 infection, one presented 6A and 6C infection, and one presented two episodes of 19A bacteremia caused by serotype 19A of MLST type ST994 in both episodes. The child received 2 doses of PCV7 before the first IPD, and had a history of recurrent acute otitis media with no other additional comorbidity. The isolates were sensitive to penicillin (MIC ≤0.1 µg/mL). No further immunological tests were carried out in this child.

### IPD Caused by PCV7-Serotypes in Vaccinated Children

Ten children had received at least one PCV7 dose before the onset of IPD caused by a PCV7 serotype (Table 1, Table 2), corresponding to an estimated incidence of 0.8 cases (95% CI 0.3–1.3) per 100.000 population. No fatal cases directly related with the IPD episode were observed in these children.

**Table 2:** Invasive pneumococcal disease due to PCV7 serotypes in PCV7 vaccinated children, Denmark 2007–2010 (n = 10).

Sex	No. of PCV7 doses received before IPD	No. of days between last PCV7 dose and IPD	Clinical presentation at admission	Comorbid Conditions	Vital status	Vaccine failure (Yes/No)*
M	1	7	Sepsis	None known	alive	No
F	2	516	Bacteremia	Complement (C2) deficiency (homozygote), MBL deficiency	alive	No
F	3	536	Bacteremia, febrile leucopenia	Acute myeloid leukaemia	alive	Yes
F	1	20	Meningitis, sepsis	None known	alive	No
M	1	145	Sepsis	Acute myeloblastic leukaemia	alive	No
F	2	207	Acute otitis media, bacteremia	None known	alive	Yes
F	1	10	Arthritis	None known	alive	No
M	1	43	Sepsis, possibly otogenic focus	None known	alive	No
M	2	71	Meningitis	None known	alive	Yes
M	1	263	Bacteremic pneumonia	Multi-handicapped child	alive	No

A case of vaccine failure is defined as a child with IPD caused by any of the serotypes included in all the vaccine doses the child has received, and where one of the three following criteria are met: 1) the child is under 13 months of age at IPD onset and has received 2 doses of PCV7/PCV13 but not yet the booster dose and becomes ill more than 2 weeks after the last dose was given; 2) the child is at least 12 months old at IPD onset, completed primary vaccination before 12 months of age and becomes ill more than 1 week after administration of the booster dose 3) the child is at least 6 months old at IPD onset, received 2 doses of PCV7 as a part of the catch-up program and becomes ill more than 2 weeks after the administration of the last dose. Vital status was recorded at 30 days after IPD. None of the patients presenting IPD due to PCV7 or one of the 6 additional serotypes in PCV13 were vaccinated with PCV13.

Seven children were considered to be incompletely vaccinated before IPD onset and did not fulfill the criteria of vaccine-failure. Among them, three children presented important comorbid conditions (Table 2). Three other cases fulfilled the criteria of vaccine failure, two following the second dose and one following the booster dose:

The first case identified as vaccine failure was observed in a 30 month old girl, who presented with pneumococcal bacteremia caused by serotype 14 after she had received a complete immunization with PCV7 at the age of 3, 5 and 12 months. The isolate was susceptible to penicillin. The child was severely immunocompromised at IPD onset; she was admitted to the hospital with febrile leukopenia after chemotherapy due to an acute myeloid leukemia (neutrophil count <200/mm3). The child recovered well after IPD and at 12-months follow-up the child did not present signs of relapse. Due to the critical clinical condition of the child and the severe immunosuppression at IPD onset, no assays for pneumococcal antibodies or additional immunological investigation were conducted.

The second case of vaccine failure was observed in a 12-month old girl, who received primary immunization with PCV7 at 4 and 6 months of age. The child was admitted to the hospital due to febrile illness, blood cultures demonstrated *S. pneumoniae* serotype 19F, susceptible to penicillin. The child was discharged from the hospital shortly after admission with the clinical diagnosis of acute otitis media and received phenoxymethylpenicillin for oral treatment. The child was previously healthy, and had no clinical history of recurrent infections. The child did not have specific testing for pneumococcal antibodies or immunologic deficiencies conducted after the clinicians' consideration.

The third case of vaccine failure was observed in an 8-month old boy, who presented with pneumococcal meningitis caused by serotype 23F after 2 doses of PCV7 given at 3 and 5 months of age. The isolate was sensitive to penicillin. The child was considered as immune competent by the clinicians in charge of the patient. The full blood count including white blood cells differential, the levels of immunoglobulins (IgG, IgA, and IgM), the Mannose-Binding lectin level, and the antibody titers to tetanus, *H. influenzae* type b, and diphtheria toxin were within normal ranges. The antibody response to PCV7 and OPA profiles are shown in Table 3. The GMC for all PCV7 serotypes but 23F were over the protective threshold, for 23F the concentration was below the threshold (0.06 µg/mL). The OPA analysis confirmed that there was incomplete (poor) killing activity for 23F. On the follow-up after 6 months the child was clinically well with no signs or symptoms of underlying medical conditions, no other episodes of invasive bacterial disease and no evidence of recurrent respiratory tract infections. The child recovered from the episode without neurological sequelae.

**Table 3:** IgG antibody concentrations and opsonophagocytic activity with type-specific polysaccharide in a child with PCV7-failure.

*Serotype*	ELISA (µg/ml)	Opsonophagocytic activity (GMT)	% inhibition*
***4***	4.098	2697	>90
***6B***	6.470	2288	>90
***9V***	3.368	2776	>90
***14***	6.304	1532	>90
***18C***	2.327	1091	>90
***19F***	5.993	3767	>90
***23F***	0.062	482	NR

The child presented with meningitis caused by serotype 23F after receiving 2 doses of PCV7. A serotype specific antibody value of 0.35 µg/mL was considered protective.

* Percent inhibition of opsonophagocytic activity after addition of type-specific polysaccharide. NR: no result given incomplete (poor) killing of the sample.

## Discussion

Results from our population-based cohort study support that PCV7 has been effective in preventing IPD caused by vaccine serotypes in the context of a 2+1 PCV program in Denmark. PCV7-type IPD in <5 year-olds occurred mainly in unvaccinated children, with only seven cases in those who received at least one dose of PCV7 (less than 1 case per 100.000 person-years) and only three cases of true vaccine failures due to serotypes 14, 19F and 23F. Among these cases, one case of vaccine failure was observed in a severely immunosuppressed child following three PCV7 doses, and two cases were observed in immunocompetent children following two infant doses before they were eligible for their booster. In addition, none of the IPD cases caused by the additional PCV13 serotypes had been observed in children vaccinated with PCV13. Thus no PCV13-vaccine failures have been observed in the first 8-months after PCV13 was started to be used in Denmark.

Concomitantly with the decrease of PCV7 serotypes observed shortly after PCV7's introduction [2], the six additional serotypes included in PCV13 were found to be the predominant cause of pediatric IPD in our cohort of patients, similarly to what has been reported from other countries [28]. However, following the introduction of PCV13 in the program, it is not clear from our data whether further benefit in preventing IPD in children under 5 years could potentially be achieved by including additional serotypes in a pediatric vaccine, since no additional serotypes have been shown to be clearly dominating the pneumococcal population causing replacement disease. This observation could be both related to differences in the invasive potential of pneumococci and characteristics of the population [29], [30]. Nevertheless, the surveillance period after PCV7/PCV13 introduction is relatively short (8 months), and serotype replacement has to be closely followed in the future in order to evaluate replacement disease in children.

During the surveillance period immediately following the introduction of PCV7, we found that nearly one-fourth of IPD cases (n = 45/191) were caused by a serotype included in PCV7, but only 10 children were partially or completely vaccinated with PCV7 before IPD, and only 3 cases fulfilled criteria of vaccine failure (Table 1, Table 2). Comorbid conditions were identified in one-third of vaccinated children with IPD due to PCV7 serotypes, regardless of the number of doses administered (Table 2). Similar results have been reported from the US, where children belonging to moderate and high-risk groups are recommended to receive PCV-vaccination [28]. We have previously reported an approximated 8% prevalence of comorbid conditions in a nationwide cohort of patients with pediatric IPD in Denmark [20]. However, the methods used for collecting data on comorbidity and estimating the prevalence of such conditions differ greatly between the two studies (by calculation of the Charlson index vs. telephonic interviews with physicians), which make direct comparison of the findings difficult. In any case, our findings underline the importance of continued vigilance for pneumococcal disease in febrile children with comorbidity or other risk factors that make them more susceptible to the disease even after immunization with PCV.

The first case identified as a vaccine failure was due to a serotype 14 infection, a serotype which has been otherwise considered to be quite immunogenic [11], [14], [31]. A case of serotype 14-vaccine failure has previously been described in a patient enrolled in the efficacy trial carried out among Navajo and White Mountain Apache children in the US [14]. In contrast to this case, our patient was severely immunosuppressed before IPD onset. In the case from the US, the child was not known to have underlying medical conditions and had a high concentration of antibodies against serotype 14, which were also found to be functional in the OPA assay. The authors suggest that serotype-specific correlates of protection based on the ELISA cut-off values from population-based vaccine efficacy studies are not necessarily adequate to predict the level of protection at the individual level. Due to the critical clinical condition of the patient in our cohort, it was considered inappropriate to collect samples for immunological testing in this patient and they would probably be of limited value.

Serotype 19F has been found to be the most frequent serotype causing pneumococcal meningitis in PCV7-vaccinated children in France [32]. In spite of its good immunogenicity, serotype 19F has been described to induce antibodies of lower avidity that decline faster compared to other antibodies induced by the vaccine [31]. A case of vaccine failure due to serotype 19F has previously been described in the literature, in a child who presented with pneumococcal meningitis in spite of three PCV7 doses and who was considered immunocompetent [15]. In our case, the clinical course was not severe; the child was discharged shortly after admission to the hospital with oral antibiotics. The clinicians decided not to investigate the antibody profile after IPD and had no clinical suspicion of immunosuppression in this patient.

Although the infant we described with 23F vaccine failure had received only 2 doses of PCV7, the antibody response to all the vaccine serotypes except serotype 23F was satisfactory, both measured by ELISA and OPA assay (Table 2). The child was considered to be immunocompetent. A lower vaccine response to this serotype has previously been reported, with the protective threshold for antibodies to this serotype (alike serotype 6B) being reached only after the booster dose [12].

Recommendations regarding clinical management and revaccination of children presenting vaccine failures must be tailored to the individual child in different clinical settings. Probably, an additional vaccine-dose followed by antibody responses and OPA measurements are suitable recommendations, since it is well documented that in some cases of vaccine failures the patient remains refractory to responses to the invading serotype despite further doses after the infection [5], [29], [31].

Concerning IPD caused by PCV7 serotypes in non-vaccinated patients, we found that nearly 40% of children were eligible for vaccination but presented with IPD before the first dose, mainly children who were targeted by the catch-up program. It has also been described that the coverage of PCV7 is slightly lower than the coverage of DTaP-IPV/Hib, and particularly for the catch-up program. The reasons for this difference are currently unknown [1]. Still, PCV7's uptake achieved in Denmark is one of the highest in Europe, where large differences in PCV7 vaccination schemes and use of PCV7 between regions have been reported [33].

Among the strengths of our study, we can mention that we were able to report high-quality data collected through an enhanced surveillance system based on collaboration between NSR, the Department of Epidemiology, the departments of clinical microbiology and clinicians across the country (Figure 1). The register of pediatric IPD due to PCV7 serotypes or to one of the six serotypes included in PCV13 includes complete clinical, epidemiological and laboratory information on patients, which will allow us to closely monitor and improve our understanding on cases of vaccine failure. Among the needs and opportunities for our surveillance system in the future we envisage the collection of information on vaccination history from IPD-cases due to other serotypes than those included in the vaccines. This would allow us to investigate whether certain groups of children are at higher risk of getting IPD and directly assess the effectiveness of the vaccines using the indirect cohort method [5], [34].

The results of our study may be limited by several factors. We present the results from a relatively short surveillance period (39 months) which followed the introduction of PCV7 in the immunization program. This may be of importance in terms of evaluation of replacement disease among vaccinated children, considering that pneumococcal populations are highly dynamic over time and susceptible to external factors such as conjugate vaccination pressure [35]. Also, children in the study population have recently been vaccinated with PCV and a large part of the population is still incompletely vaccinated, limiting the extrapolation of these results in the long term.

A selection bias may also be present in our study, since we used a highly specific case definition including only culture confirmed IPD cases in a setting were blood- and CSF-cultures are almost exclusively obtained in hospital settings. Furthermore, we were not able to investigate the immunological status after IPD in all children with vaccine failure, which could have contributed to the understanding the underlying mechanisms.

### Conclusions

In summary, IPD in children younger than 5 years of age was a rare event and occurred mainly in unvaccinated children in our cohort of patients. Only three cases of PCV7-failure were confirmed during a 31-month surveillance period. Seven additional IPD cases due to PCV7- serotypes were observed in incompletely vaccinated children. So far, no PCV13-vaccine failure cases have been observed, however the surveillance period was short. Children with confirmed or suspected immunosuppression should be closely monitored and assessed on an individual basis.
